# Neural responses to children’s faces: Test–retest reliability of structural and functional MRI

**DOI:** 10.1002/brb3.1192

**Published:** 2019-02-09

**Authors:** Esther Heckendorf, Marian J. Bakermans‐Kranenburg, Marinus H. van Ijzendoorn, Rens Huffmeijer

**Affiliations:** ^1^ Department of Education and Child Studies Leiden University Leiden The Netherlands; ^2^ Leiden Institute for Brain and Cognition (LIBC) Leiden University Leiden The Netherlands; ^3^ Clinical Child and Family Studies Vrije Universiteit Amsterdam The Netherlands; ^4^ Department of Psychology, Education and Child Studies Erasmus University Rotterdam The Netherlands

**Keywords:** face processing, fMRI, handedness, structural MRI, test‐retest reliability

## Abstract

**Introduction:**

Functional MRI (fMRI) is commonly used to investigate the neural mechanisms underlying psychological processes and behavioral responses. However, to draw well‐founded conclusions from fMRI studies, more research on the reliability of fMRI is needed.

**Methods:**

We invited a sample of 41 female students to participate in two identical fMRI sessions, separated by 5 weeks on average. To investigate the potential effect of left‐handedness on the stability of neural activity, we oversampled left‐handed participants (*N* = 20). Inside the scanner, we presented photographs of familiar and unfamiliar children's faces preceded by neutral and threatening primes to the participants. We calculated intraclass correlations (ICCs) to investigate the test–retest reliability of peak activity in areas that showed significant activity during the first session (primary visual cortex, fusiform face area, inferior frontal gyrus, and superior temporal gyrus). In addition, we examined how many trials were needed to reliably measure the effects.

**Results:**

Across all participants, only fusiform face area activity in response to faces showed good test–retest reliability (ICC = 0.71). All other test–retest reliabilities were low (0.01 ≤ ICC ≤ 0.35). Reliabilities varied only slightly with increasing numbers of trials, with no consistent increase in ICCs. Test–retest reliabilities for left‐handed participants (0.28 ≤ ICC ≤0.66) were generally somewhat higher than for right‐handed participants (−0.13 ≤ ICC ≤0.75), but not statistically significant.

**Conclusion:**

Our study shows good test–retest reliability for fusiform facer area activity in response to faces, but low test–retest reliability for other contrasts and areas.

## 1 INTRODUCTION

Twenty‐five years after the first functional MRI (fMRI) experiment was conducted (Belliveau et al., 1991), fMRI has grown into a universally used method to study the neural correlates of both psychological and behavioral responses to visual or auditory stimuli. However, firm conclusions can only be drawn from fMRI experiments if the measurements are valid, that is, assess what they are supposed to measure, and test–retest reliable, that is, provide stable results over time. Reliability is usually considered a prerequisite for validity (Feldt & Brennan, 1989; Gay, 1987).

In earlier studies, fMRI test–retest reliability was investigated using various tasks and experimental designs (Bennett & Miller, 2010; Herting, Gautam, Chen, Mezher, & Vetter, 2017). Bennett and Miller (2010) computed an average intraclass correlation coefficient (ICC) of 0.50 across 13 earlier fMRI reliability studies, but report substantial variation across studies, with ICCs ranging from 0.16 to 0.88. Similarly, Herting et al. (2017) reviewed test–retest reliabilities for 12 longitudinal task‐based fMRI studies with children and adolescents. ICC values varied between poor and excellent, depending on the specific task and region of interest (ROI) examined. Thus, the considerable variance in ICC values for task‐related fMRI measures may be caused by technical factors (e.g., magnet strength of the scanner), the brain area and process under investigation (e.g., visual processing, memory), task design (e.g., block design vs. event‐related design), sample characteristics, and the time interval between the two assessments (Bennett & Miller, 2010; Herting et al., 2017). In the current study, we investigated the influence of some of these factors on test–retest reliability in a face processing paradigm.

A Web of Science search with the search terms “face” and “fMRI” (WoS, 1 September 2016) results in more than 4,000 hits for studies conducted during the last 20 years, which illustrates how common the investigation of face processing in neuroimaging research is. Nevertheless, studies assessing test–retest reliability for face processing tasks are surprisingly rare. The existing studies of test–retest reliability of fMRI activity in face processing paradigms focused on the processing of faces with emotional expressions. Three of these studies reported poor test–retest reliability of amygdala activity (Lipp, Murphy, Wise, & Caseras, 2014; Plichta et al., 2012; Sauder, Hajcak, Angstadt, & Phan, 2013; Van den Bulk et al., 2013). In the other three studies, reliability estimates of amygdala activity varied from fair to excellent (Cao et al., 2014; Gee et al., 2015; Schacher et al., 2006). Test–retest reliability for regions other than the amygdala revealed fair reliability for prefrontal cortex activity (Van den Bulk et al., 2013), fair to good test–retest reliability for fusiform face area (FFA) activity (Sauder et al., 2013), and fair to excellent reliability for the inferior frontal gyrus (IFG), anterior cingulate gyrus (ACC), and fusiform gyrus (Gee et al., 2015). The sample sizes of these studies were mostly small, ranging from 8 to 27 participants. In fact, neuroscientific studies tend to be underpowered in general (due to small sample sizes and/or small effects; Button et al., 2013), and fMRI reliability studies are no exception. In the review of Bennett and Miller (2010), the overall sample size across 63 studies was 11, with many studies using fewer than 10 subjects for reliability measures. fMRI reliability studies with larger sample sizes are thus badly needed. Here, we aimed to fill this gap by conducting a reliability study with a larger sample (*N* = 41).

Moreover, to the best of our knowledge, no studies have yet investigated the test–retest reliability of a face processing paradigm with faces with neutral expressions only, although these are regularly used in fMRI research. Here, we specifically address the test–retest reliability of fMRI activity during a face processing task with faces without emotional expressions that can be used to study adults’, including parents’, neural responses to (their own) children's faces (Heckendorf, Huffmeijer, Bakermans‐Kranenburg, & van IJzendoorn, 2016). We examine the test–retest reliability of fMRI data acquired during two sessions separated by a period of 4–12 weeks. Because the stability of significant activity is particularly informative in light of the reproducibility of neuroimaging research, we computed between‐session reliability of effects that were significant in session 1 (for details see Heckendorf et al., 2016). We targeted the following regions of interest (ROIs): IFG, superior temporal gyrus (STG), fusiform face area (FFA), and primary visual cortex (V1). However, as limited reliability within a single session may negatively affect test–retest reliability, we also computed within‐session reliability for both session 1 and session 2. Based on the meta‐analysis of Bennett and Miller (2010), we expected fair test–retest reliability values for the fMRI data in our study.

We examined effects of two specific factors on reliability. First, we examined whether test–retest reliability differs between left‐ and right‐handed participants. Left‐handers are frequently excluded from neuroimaging studies to prevent the introduction of unwanted noise in group statistics that would, for instance, be caused by potential differences in lateralization between left‐ and right‐handers (Willems, Van der Haegen, Fisher, & Francks, 2014). However, about 10% of humans are left‐handed, and thus, left‐handers represent a significant proportion of the human population (McManus, 2009). Thus, we aim to examine to what extent brain activity of left‐handed participants can be measured as reliably as right‐handed participants’ brain activity. Second, we examined the influence of task length, and thus the number of volumes scanned per participant. In resting‐state fMRI, both increasing the number of volumes and increasing the time over which these volumes are acquired have been shown to improve within‐ and between‐session reliabilities (Birn et al., 2013). Likewise, in ERP‐studies, the reliability of averaged ERPs can benefit from increasing the numbers of trials (Huffmeijer, Bakermans‐Kranenburg, Alink, & van Ijzendoorn, 2014). Whether increasing the number of trials of a task significantly improves test–retest reliability of task fMRI data has not yet been studied systematically.

Finally, we assessed test–retest reliability for several measures of structural MRI as a comparison to fMRI. We focused on measures of gray and white matter volume as well as volumetric measures of two subcortical structures: the amygdala and the thalamus. We expected good to excellent reliability of all volumetric measures, in accordance with earlier research (Bartzokis et al., 1993; Convit et al., 1999; Morey et al., 2010).

## 2 MATERIALS AND METHODS

### 2.1 Participants

We invited 49 female undergraduate and graduate students with an average age of 21.73 years (*SD* = 2.55, range 18–28 years) for two experimental sessions, 4–12 weeks (*M* = 4.61, *SD* = 1.68 weeks) apart. Exclusion criteria were MRI contraindications, pregnancy, current psychiatric and neurological disorders, severe head injury, current alcohol or drug abuse, and chronic use of medication (except contraceptives). Data of two of the participants could not be included in test–retest reliability calculations, because they only completed the first session of the experiment. In addition, data of six participants were excluded from analyses because of excessive head movements (>3 mm; *n* = 2) or falling asleep during the fMRI recording (*n* = 4). Thus, our final sample consisted of 41 participants aged 21.81 years on average (*SD* = 2.67; range 18–28 years). The Ethics Committee of the Leiden University Medical Center approved the study and all participants signed informed consent at the beginning of the first session. Participation was rewarded with 40 €. All participants’ structural scans were evaluated by a radiologist employed by the Leiden University Medical Centre, and no anomalies were found.

### 2.2 Procedure

Prior to the first session, participants’ completed Van Strien's (1992) 10‐item Handedness Questionnaire, which measures hand preference during execution of several tasks (e.g., “Which hand do you use to brush your teeth?”). Items are scored on a 3‐point scale (left hand, both hands, right hand) ranging from −1 to 1. Total scores can thus vary between −10 and +10. Based on their scores, we divided the participants into two groups: participants with a score of +1 or higher were defined as right‐handed (*N* = 21), and participants with a score of −1 or lower were classified as left‐handed (*N* = 20). We oversampled left‐handed participants to investigate the potential effect of left‐handedness on the stability of neural activity.

**Figure 1:**
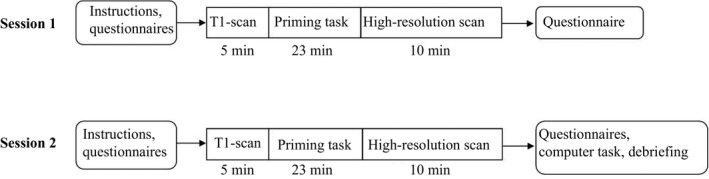
Schematic overview of session 1 and session 2 and the scan procedures. Scan procedures were identical for the two sessions

We asked participants to abstain from alcohol and excessive physical activity during the last 24 hr and from caffeine during the last 12 hr before the start of each session. In session 1, participants filled out the Children's Report of Parental Behavior Inventory (CRPBI‐30, Schludermann & Schludermann, 1983; Beyers & Goossens, 2003) and the Interpersonal Reactivity Index (De Corte et al., 2007; Davis, 1980).Results relating to these questionnaires and fMRI data obtained during the first session have been reported elsewhere (Heckendorf et al., 2016). At the beginning of each session, the MRI procedure was explained to the participants. Inside the scanner, foam inserts were placed between the head coil and the participant's head to minimize head movements. Within the scanner, participants completed a priming task (see below), during which visual stimuli were projected onto a screen placed outside the opening of the scanner bore. Participants viewed the screen through a mirror fixed to the head coil. At the end of the second session, participants completed a task in which they judged several characteristics of various faces (data to be reported elsewhere). Subsequently, participants were debriefed about the nature of the priming task. Figure 1 shows a schematic overview of the procedures in each session.

### 2.3 Experimental task

**Figure 2:**
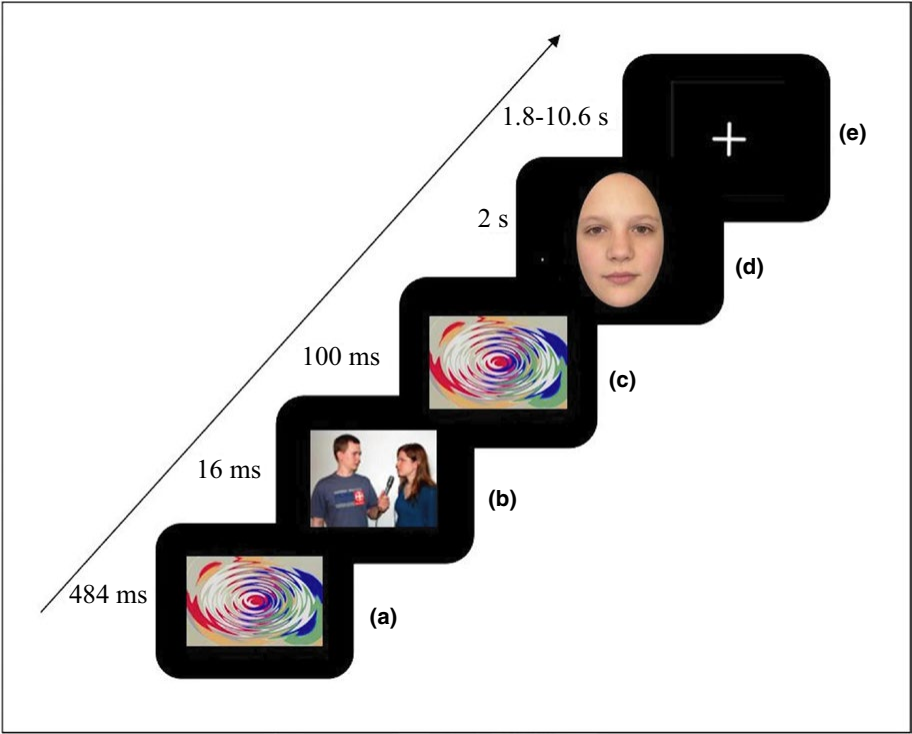
The priming task. A neutral or threatening prime (b) was presented for 16 ms on the screen, concealed by a mask presented immediately before (a) and after (c) the prime. The second mask was followed by an unfamiliar‐looking, a familiar‐looking or a scrambled face (d). During intertrial intervals, a fixation (e) cross was presented

Inside the scanner, subjects completed a priming task consisting of 234 trials. The priming task was set‐up in an event‐related design. All stimuli were shown in the center of the screen on a black background. On all trials, a colored, circular pattern was used for forward and backward masking of the primes to prevent conscious perception of the primes. The mask was matched for size and average luminosity of the primes. During each trial, a fixation cross was presented (1,800–10,600 ms), followed by the mask (presented for 484 ms), a neutral or a threatening prime (presented for 16 ms), again the mask (presented for 100 ms) and an unfamiliar‐looking, a familiar‐looking or a scrambled face (presented for 2,000 ms). Thus, the priming task consisted of six conditions: a familiar‐looking face presented after a neutral prime (neutral–familiar), a familiar‐looking face presented after a threatening prime (threat–familiar), an unfamiliar‐looking face presented after a neutral prime (neutral–unfamiliar), an unfamiliar‐looking face presented after a threatening prime (threat–unfamiliar), a scrambled face presented after a neutral prime (neutral–scrambled), and a scrambled face presented after a threatening prime (threat–scrambled). We presented stimulus sequences (mask‐prime‐mask‐[scrambled]face) in quasi‐random order with the following restrictions: The same prime could not be presented more than twice in a row, the same face could not be presented more than four times in a row, and the same condition could not be presented more than two times in a row. In total, the priming task consisted of 13 neutral and 13 threatening primes that were each presented three times with each face, resulting in 39 (3*13) trials per condition. Participants had to press a button every 11–13 trials to continue the task to verify that they remained alert. On average, the task took 23 min. Figure 2 illustrates a trial of the priming task.

### 2.4 Primes

In previous research, Nummenmaa, Hirvonen, Parkkola, and Hietanen (2008) developed pairs of neutral and threatening photographs that were matched on luminosity, global energy, contrast density, and complexity, and depicted two persons in comparable proximity to each other. Threatening scenes portrayed interpersonal attack scenes (e.g., one person strangling the other), whereas neutral scenes depicted emotionally neutral situations (e.g., two persons having a conversation). We used these neutral and threatening photographs as primes for our study with the objective to investigate subliminal processing of neutral and threatening stimuli. For a detailed description concerning the selection of the neutral and threatening pairs and the visibility of the primes in our study, see Heckendorf et al. (2016).

### 2.5 Facial stimuli

We morphed a photograph of a child's face (unfamiliar to the participant) with (a) a photograph of an unknown female's face and (b) a photograph of the participant's own face to create unfamiliar‐ and familiar‐looking children's faces. For this purpose, we asked participants prior to the first session to provide a full color digital photograph of themselves that met the following criteria: picture on a light and uniform background, showing their face (full frontal) and neck only, with a neutral facial expression, and no piercings, make‐up or glasses. To create the unfamiliar‐looking morphs, we used two full color, full frontal photographs of two female Caucasian faces (aged 24 and 25 years) with neutral facial expression, no jewelry or glasses and unfamiliar to the participant. For half of the participants, we created unfamiliar‐looking morphs with female face 1 for session 1 and female face 2 for session 2, and for the other half vice versa. Full color, full frontal photographs of six 9‐ to 11‐year‐old children (three boys and three girls, all Caucasian [but slightly varying in skin color], all unfamiliar to the participants, with neutral facial expression, no jewelry or glasses) were available for morphing. We used a picture of a female child to create morphs for half of the participants and a picture of a male child to create morphs for the other half of the participants. Within genders, the child that best matched the participant's skin color and face‐shape was selected for ease of morphing. We used the photograph of the same child to create unfamiliar‐looking and familiar‐looking morphs for a participant. One familiar‐looking and two unfamiliar‐looking morphs were created for the two sessions, because using the same unfamiliar‐morph for both session would have increased familiarity with the unfamiliar‐looking face in session 2 compared to session 1. Using a different unfamiliar‐looking face in session 2 ensured that participants’ familiarity with the unfamiliar‐looking face was kept constant across sessions, in order to avoid effects on test–retest reliability. To generate the morphs, all photographs were first resized to 448 × 560 pixels and edited using Adobe Photoshop CS: External features (i.e., hair and ears) were removed and the pictures were pasted on a black background. Next, Fantamorph 5 Deluxe was used to create the morphs. We created familiar‐looking morphs that consisted for 50% of the participant's face and for 50% of an unknown child's face, and unfamiliar‐looking morphs that consisted for 50% of the unknown female's face and for 50% of the child's face. The resulting morphs looked somewhat older than the 9‐ to 11‐year‐olds used for morphing and appeared to be about 14 years old (see Heckendorf et al., 2016). Finally, a scrambled face was created for each participant from the familiar‐looking morph by randomly rearranging blocks of 9 × 9 pixels using Matlab R2012B.

### 2.6 Image acquisition

Images were acquired at the Leiden University Medical Center on a 3‐T Philips Achieva MRI system (Philips Medical Systems, Best, Netherlands) with a 32‐channel SENSE (Sensitivity Encoding) head coil. An event‐related design with 680 T2*‐weighted whole‐brain echo planar images (EPI, repetition time [TR] = 2,200 ms, echo time [TE] = 30 ms, flip angle = 80°, 38 transverse slices, descending acquisition order, voxelsize = 2.75 × 2.75 × 3.025 mm^3^ with a 10% interslice gap, field of view [FOV] = 220 × 114.675 × 220 mm^3^) was used for the functional scans. To avoid magnetic saturation effects, the first four functional scans were discarded. In addition, an anatomical 3D T1‐weighted scan (TR = 9.825 ms, TE = 4.605 ms, inversion time [TI] = 1,050 ms, shot interval = 1,932 ms, flip angle =8°, 140 transverse slices, voxelsize 0.875 × 0.875 × 1.2 mm^3^, FOV = 224 × 168 × 177.333 mm^3^) and a high‐resolution T2*‐weighted EPI (TR = 2,200 ms, TE = 30 ms, flip angle = 80°, 84 transverse slices, voxel size = 1.964 × 1.964 × 2 mm^3^, FOV = 220 × 168 × 220 mm^3^) were obtained during each session for coregistration.

### 2.7 fMRI data analysis

Data analyses were carried out using FSL (FMRIB's Software Library1) FEAT (FMRI Expert Analysis Tool) version 5.0.4, part of Jenkinson, Beckmann, Behrens, Woolrich, and Smith (2012) and Smith et al. (2004). Data obtained during sessions one and two were processed identically. Because we were interested in potential effects of task length on the reliability of MRI data, and in within‐session reliability, analyses were performed on both the complete datasets and several subsets of data: (a) data obtained during the first third (78 trials) and (b) first two thirds (156 trials) of the task, as well as data collected during (c) the second third and (d) final third of the task only. We made use of the Fslroi toolbox of FSL (FMRIB's Software Library) to create the different subsets of the data. Subsequently, data of the different subsets were processed identically to the data of the whole task.

Four prestatistics processing steps were applied to the data: motion correction (MCFLIRT; Jenkinson, Bannister, Brady, & Smith, 2002), non‐brain removal (using BET; Smith, 2002), spatial smoothing using a Gaussian kernel with a full‐width‐at‐half‐maximum of 6 mm, and high‐pass temporal filtering with a high‐pass filter cutoff of 100 s. Subsequently, functional images were registered to the high‐resolution EPI, which was then registered to the 3D T1‐weighted scan, and then to the 2 mm isotropic MNI‐152 standard space image (T1 standard brain averaged over 152 subjects; Montreal Neurological Institute, Montreal, QC, Canada; Jenkinson et al., 2002). Functional images of session 1 were registered to the high‐resolution EPI and the 3D T1‐weighted scan of session 1. Functional images of session 2 were registered to the high‐resolution EPI and the 3D T1‐weighted scan of session 2. General linear model analyses in native space were performed to examine functional activity in response to the stimuli. Because primes and masks were displayed on the screen for very short durations and time‐locked to the presentation of the faces, hemodynamic responses to the individual stimuli within a mask‐prime‐mask‐face sequence overlapped substantially and summed to a total, summed hemodynamic response to the stimulus sequence. Hence, we treated the presentation of a mask‐prime‐mask‐face sequence as a single stimulation period. Thus, we modeled the different conditions (threat–familiar, threat–unfamiliar, threat–scrambled, neutral–familiar, neutral–unfamiliar, and neutral–scrambled) and participants’ button press responses as seven explanatory variables using the Custom (three column format) wave function convolved with a double gamma hemodynamic response function. The temporal derivatives of the explanatory variables were included in the model, yielding 14 regressors.

As described in Heckendorf et al. (2016), ROI‐ and whole‐brain analyses of session 1 revealed greater activity in the primary visual cortex (V1) in all conditions of the priming task (threat–familiar, threat–unfamiliar, threat–scrambled, neutral–familiar, neutral–unfamiliar, neutral–scrambled) compared to fixation cross. Additionally, compared to unfamiliar faces, familiar faces evoked enhanced activity in the right IFG and in bilateral FFA, and unfamiliar faces, compared to familiar faces, elicited increased activity in bilateral STG. ICC values can be affected by systematic differences in brain activity between the sessions. Thus, to identify a possible session effect, we conducted separate ROI‐ and whole‐brain analyses in which we compared activity in session 1 with activity in session 2 (for this purpose, we added a comparison of the two sessions to the model described in Heckendorf et al., 2016). The whole‐brain and ROI analyses did not reveal any significant session effects.

To analyze test–retest reliability of activity within the brain areas showing significant effects in session 1, we created two types of ROI‐masks: a mask matching the area showing significant activity (differences) in session 1 (functional mask) and an a priori‐defined mask. A priori‐defined masks for the IFG and STG were defined anatomically using the Harvard‐Oxford Cortical Structures Atlas. For V1, a priori‐defined mask was defined anatomically using the Juelich Histological Atlas (both Atlases are implemented in FSL version 5.0.4). Three binarized, a priori‐defined masks consisting of voxels belonging to V1, left or right IFG and STG, respectively, with a probability of at least 25% were created in 2 mm isotropic MNI‐152 standard space (Jenkinson et al., 2002). As the FFA is an area within the fusiform gyrus defined by its preferential responding to faces, we first created a mask of the FFA using the probability map obtained for a localizer task in an earlier study (*N* = 124) for the contrast faces versus scenes (Engell & McCarthy, 2013). Subsequently, we binarized and thresholded this contrast image (only voxels with a 25% probability to be significantly activated in the faces vs. scenes contrast included) in 2 mm isotropic MNI‐152 standard space (Jenkinson et al., 2002). Next, we defined a mask of the fusiform gyrus using the Harvard‐Oxford Cortical Structures Atlas, including only voxels belonging to the right of left fusiform gyrus with a probability of at least 25% in 2 mm isotropic MNI‐152 standard space (Jenkinson et al., 2002). Finally, we multiplied this mask with the thresholded face versus scene contrast image to obtain a priori‐defined mask for the FFA.

**Figure 3:**
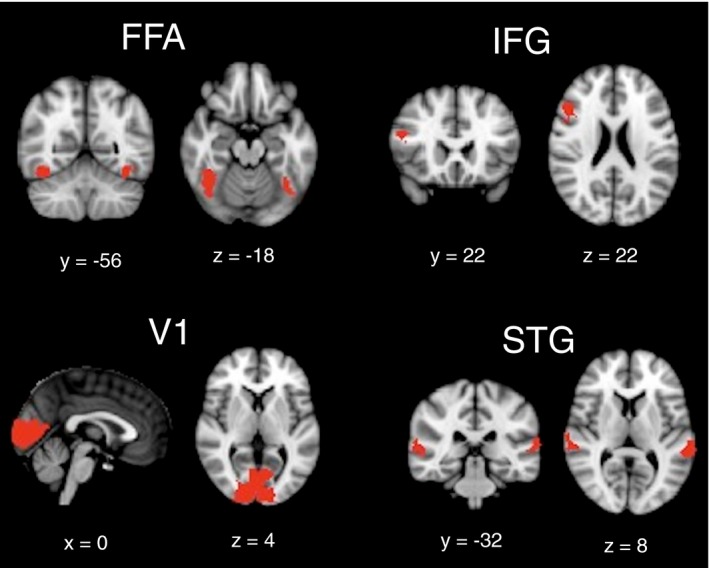
Functional masks of the fusiform face area (FFA), primary visual area (V1), inferior frontal gyrus (IFG), and superior temporal gyrus (STG)

To create functional masks, we binarized and thresholded (*Z* > 2.3) the contrast images of significant effects obtained in session 1, using Fslstats (FMRIB's Software Library). Next, we multiplied these thresholded contrast images with a priori‐defined masks that we had created before (as significant clusters of activity sometimes extended over several anatomical areas or, conversely, covered only a part of the anatomical region). This resulted in the following four functional masks: (a) IFG and (b) STG masks matching activity obtained for the contrast familiar versus unfamiliar, (c) FFA mask matching the combined activity obtained for the contrasts familiar versus unfamiliar and face versus scrambled, and (d) V1 activity obtained for the combined activity of the different conditions of the priming task versus fixation cross. The functional masks used to examine the test–retest reliability in the different ROIs are presented in Figure 3.

Subsequently, we used Featquery (Smith et al., 2004) to extract participants’ mean, median, and maximum cope values (contrast of parameter estimates, i.e., beta weights [for activity within a single condition] or differences between beta weights [for contrasts between conditions]) for each ROI and contrast of interest. These values were exported to IBM SPSS Statistics 23 for further analysis. As the reliability of significant activity was our main interest, results for functional masks are presented in the results section and results for a priori‐defined masks can be found in the Supporting information.

### 2.8 Structural MRI analyses

The anatomical 3D T1‐weighted scans of session 1 and session 2 were segmented into gray matter, white matter, and cerebrospinal fluid using FSL (FMRIB's Software Library) FAST (fMRI Automated Segmentation Tool) version 5.0.4, part of Jenkinson et al. (2012) and Smith et al. (2004). We used the Fslstats toolbox of FSL (FMRIB's Software Library) to extract measures of gray and white matter volume (in ml) from the partial volume maps that were created with FAST. We segmented the left and right thalamus and amygdala using FIRST (FMRIB's integrated registration and segmentation tool) and extracted measures of tissue volume (ml) of the left thalamus, right thalamus, left amygdala, and right amygdala using Fslstats. All values were exported to IBM SPSS Statistics 23 for further analysis. For one participant who was included in the fMRI test–retest reliability analyses, only the 3D T1‐weighted scan of session 2 was available (due to a large artifact on the scan from session 1), and thus, it was not possible to calculate test–retest reliability of the structural MRI data for this participant. For this participant, the 3D T1‐scan of session 2 was used for the registration of the functional images of both session 1 and session 2.

### 2.9 Intraclass correlation

To investigate test–retest reliability, we calculated ICCs (2‐way mixed model, single measures, absolute agreement) between values (volumetric measures for structural MRI and copes for fMRI data) obtained during sessions 1 and 2. According to Cicchetti (2001), ICC values below 0.40 indicate poor reliability, values between 0.40 and 0.59 fair reliability, values between 0.60 and 0.74 good reliability, and values above 0.74 indicate excellent reliability.

We calculated ICCs for the following values: (a) gray and white matter volumes, and tissue volumes of left thalamus, right thalamus, left amygdala, and right amygdala obtained from structural MRI data, and (b) mean, median, and maximum values within the IFG, FFA, and STG for the contrast familiar versus unfamiliar, within the FFA for the contrast face versus scrambled, and within V1 for the different conditions of the priming task versus fixation cross. In general, ICCs were largest for maximum values from functional ROIs. Therefore, we present only ICCs for the maximum values in the results section (results for mean and median values are presented in the Supporting information), and focus on ICCs for maximum values in analyses of potential effects of task length and handedness. To investigate potential effects of the number of trials included, we calculated ICC values for different subsets of the data. For this purpose, we compared ICC values of the first third (78 trials), the first two thirds of the task (156 trials), and the complete task (234 trials). Finally, to investigate potential effects of handedness on test–retest reliability, we computed ICCs (complete task) for left‐ and right‐handed participants separately. We computed Fisher's *r* to *z* transformation to examine potential effects of handedness. To control for multiple testing, we applied the Benjamini–Hochberg procedure (Benjamini & Hochberg, 1995). For the structural data, we controlled for the number of tests conducted to examine test–retest reliability of volumetric measures for structural MRI. For functional data, we applied the Benjamini–Hochberg procedure separately for each of the functional processes that we investigated (visual processing [stimuli vs. fixation cross in V1], processing of familiarity [contrast: familiar vs. unfamiliar in IFG, STG, and FFA], and face processing [contrast: face vs. scrambled in FFA]).

In addition to ICCs for absolute agreements, we also computed ICCs for consistency (two‐way mixed model, single measures, consistency) for maximum values from functional ROIs, to ensure that our ICCs for absolute agreements were not negatively affected by systematic activity differences between the sessions. Results related to ICCs obtained for consistency are presented in the Supporting information (Table S5). We also investigated within‐session reliability. For this purpose, within‐session reliability was calculated as the ICC across values obtained during the first third, second third, and final third of the task in session 1 (each subset consisting of 78 trials). Likewise, within‐session reliability of session 2 was calculated as the ICC across values obtained during the first third, second third, and final third of the task in session 2. Results regarding within‐session reliability are presented in the Supporting information. Because deviations from normal distributions and outliers may influence the ICCs, we examined skewness and kurtosis and possible outliers (*Z* ≥ 3.29) for all distributions prior to test–retest reliability calculations. For distributions with outliers, we calculated test–retest reliability both with and without outliers. Removing outliers from the sample did not lead to substantial changes in test–retest reliabilities. In case of non‐normal distributions, we calculated both Spearman's rho and the ICC, as Spearman's rho is not affected by non‐normality. Spearman's rho did not substantially differ from the ICC. We therefore report only ICCs below.

## 3 RESULTS

### 3.1 Structural analyses

The means and standard deviations of volumetric measures in session 1 and session 2 were highly similar. Test–retest reliabilities for both cortical (gray matter and white matter) and subcortical (amygdala and thalamus) volumetric measures were excellent, both across the entire sample (0.80 ≤ ICC ≤ 0.98) and for left‐handed (0.93 ≤ ICC ≤ 0.98, with the exception of right amygdala volume ICC = 0.74) and right‐handed (0.81 ≤ ICC ≤ 0.99) participants separately. Differences between left‐ and right‐handed participants in structural test–retest reliabilities were small and not significant, as tested using Fisher's *r* to *Z* transformation (See Supporting information Table S1 for a detailed overview of ICC values).

### 3.2 fMRI test–retest reliability

Table 1 summarizes test–retest reliabilities obtained for maximum cope values. As evident from Table 1, only FFA activity related to face processing (i.e., the contrast face vs. scrambled) showed good reliability, both across the entire sample (ICC = 0.71), and for left‐handed (ICC = 0.66) participants, as well as excellent reliability for right‐handed (ICC = 0.75) participants. V1 activity showed fair reliability for left‐handed participants only (0.45 ≤ ICC ≤0.57; except for NeutralUnfamiliar, ICC = 0.35) and poor reliability for right‐handed participants (ICC ≤ 0.18) as well as across the entire sample (0.28 ≤ ICC ≤0.35). Test–retest reliability for the contrast familiar versus unfamiliar was poor in all tested ROIs (ICC ≤ 0.28) across the entire sample and for left‐ and right‐handed participants separately, except for FFA activity for left‐handed participants, which was fairly reliable (ICC = 0.46).

**Table 1:** Test–retest reliabilities for maximum values of the whole sample for the first third (78 trials *N *= 42^a^), the first two thirds of the task (156 trials *N *= 42^a^), and the complete task (234 trials *N* = 41), and for left‐ (*N* = 20) and right‐handed (*N* = 21) participants separately (234 trials)

ROI	Contrast	Number of trials			Left‐handed	Right‐handed	Fisher's *r* to *z*	
		78	156	234			*Z*	*p*
V1	ThreatFamiliar vs. fix	0.41	0.35	0.35	0.57	0.06	1.74	0.08
	ThreatUnfamiliar vs. fix	0.40	0.41	0.35	0.53	0.07	1.53	0.13
	ThreatScrambled vs. fix	0.51	0.40	0.35	0.53	0.13	1.37	0.17
	NeutralFamiliar vs. fix	0.46	0.46	0.29	0.48	0.02	1.51	0.13
	NeutralUnfamiliar vs. fix	0.44	0.41	0.28	0.35	0.18	0.56	0.58
	NeutralScrambled vs. fix	0.43	0.29	0.28	0.45	0.07	1.21	0.23
FFA	Familiar vs. Unfamiliar	0.13	0.25	0.25	0.46	0.04	1.36	0.17
	Face vs. Scrambled	0.60	0.65	0.71	0.66	0.75	−0.52	0.60
IFG	Familiar vs. Unfamiliar	0.06	0.11	0.01	0.28	−0.13	1.22	0.22
STG	Familiar vs. Unfamiliar	0.12	0.07	0.16	0.28	−0.04	0.96	0.34

Note

fix: fixation cross.

a For one participant, data were only available for the first and the second part of the task, since this participant fell asleep during the third part.

As can be seen in Table 1, there is no consistent trend for increasing ICCs with increasing numbers of trials, and reliabilities vary only slightly (0.06 ≤ ICC ≤0.60 [78 trials], ICC 0.07 ≤ ICC ≤0.65 [156 trials], 0.01 ≤ ICC ≤0.71 [234 trials]). Notably, for V1 we obtained fair reliability for all contrast when analyzing only the first third of the task (78 trials, 0.40 ≤ ICC ≤ 51), with ICCs seemingly decreasing with increasing numbers of trials, although the differences are small. As shown in Table 1, test–retest reliabilities were somewhat higher for left‐handed (0.28 ≤ ICC ≤ 0.66) than for right‐handed participants (−0.13 ≤ ICC ≤0.75), with the exception of the ICC for FFA activity in the contrast faces versus scrambled. However, the differences in test–retest reliability between left‐ and right‐handed participants, as tested using Fisher's *r* to *Z* transformation, were not statistically significant.

Results obtained for mean and median values are displayed in the Supporting information (Tables S3 and S4). Test–retest reliabilities of mean and median values were generally lower than those of maximum values but showed largely the same pattern, with reliable results obtained only for FFA activity related to face processing (face vs. scrambled). We also investigated test–retest reliabilities for maximum values within the a‐priori defined ROIs (see Supporting information, Table S2). The ICCs obtained were highly similar to those acquired for the functional masks. In addition, s5we calculated within‐session reliabilities to examine whether low reliability values might be explained by systematically low reliability in one session (see Supporting information, Table S6). For the contrast familiar versus unfamiliar, we obtained low ICCs for session 1 and session 2 for all tested ROIs. However, V1 activity for the contrasts comparing activity in response to the stimulus conditions to fixation cross, and FFA activity for the contrast face versus scrambled, tended to be more reliable in session 1 than in session 2, suggesting that some habituation may have occurred between the sessions and/or within session 2. In addition, ICCs for V1 activity were systematically higher for left‐handed than for right‐handed participants in session 2 (although significant only for the contrast NeutralUnfamiliar vs. fixation cross), but not in session 1. This mainly reflects lower within‐session reliability for right‐handed participants in session 2 when compared to session 1. In fact, reliability of V1 activity was fair to excellent within session 1 (both across the groups and for left‐ and right‐handed participants separately; ICC ≥ 0.47) and for left‐handed participants within session 2 (ICC ≥ 0.41) and poor only for right‐handed participants within session 2 (0.13 ≤ ICC ≤0.31, except NeutralScrambled vs. fixation: ICC = 0.46). Thus, the habituation effects mentioned above may, in V1, be limited to right‐handed participants.

Finally, ICCs for consistency were generally comparable to ICCs obtained for absolute agreement (see Supporting information, Table S5), with the exception of somewhat higher ICCs obtained for V1 activity for left‐handed participants for consistency compared to absolute agreement. In addition, ICCs for consistency were systematically higher for left‐handed participants than for right‐handed participants (although significant only in V1 for the contrasts ThreatFamiliar vs. fixation cross, ThreatScrambled vs. fixation cross, and NeutralUnfamiliar vs. fixation cross after correction for multiple testing).

## 4 DISCUSSION

The main purpose of this study was to assess test–retest reliability of significant fMRI activity acquired during a face processing paradigm in a priming context. Reliabilities of structural MRI data were generally excellent, with the exception of good reliability for right amygdala volume measured in left‐handed participants. Somewhat lower reliability for amygdala volumes compared to larger subcortical structures were also obtained in earlier research using the same segmentation procedure (Morey et al., 2010). The reliabilities obtained for fMRI data were generally lower than expected. Stable activity was found only for the FFA in response to familiar and unfamiliar faces compared to scrambled faces. In addition, we obtained fairly stable V1 activity in left‐handed, but not in right‐handed participants. Unexpectedly, adding more trials did not substantially increase test–retest reliability, and in V1, reliability of maximum copes even decreased from fair (ICCs ≥ 0.40 for 78 trials) to poor (ICCs ≤ 0.35 for 234 trials) with an increasing number of trials. Regarding handedness, although ICCs for left‐handed participants were generally higher than for right‐handed participants, the differences were usually not statistically significant (with four exceptions, see Supporting information). Thus, whether subtle differences in the reliability of fMRI activity exist between left‐handed and right‐handed individuals remains a topic for investigation. Finally, it is worth noting that we observed larger ICCs for maximum values of activity than for mean and median values. Thus, the results presented in the results section show a relatively optimistic picture.

The good reliability of FFA activity in response to faces compared to scrambled stimuli is in line with earlier research that demonstrated robust changes in FFA activity related to face processing (e.g., Gauthier, Skudlarski, Gore, & Anderson, 2000; Haxby et al., 2001). Our results emphasize that FFA activity reflecting face processing can be measured reliably with fMRI. To the best of our knowledge, only four other studies have investigated the reliability of FFA activity related to face processing. In the first three studies, test–retest reliabilities were fair to good (McGugin & Gauthier, 2016; Sauder et al., 2013) and fair to excellent (Nord, Gray, Charpentier, Robinson, & Roiser, 2017), similar to our reliability estimates. The fourth study, however, reported low reliability (Lipp et al., 2014). The small sample size in the Lipp et al. (2014; *N* = 14) study may explain the deviating results, as studies using small sample sizes are at greater risk of drawing incorrect conclusions.

The low reliability of IFG, STG, and FFA activity in response to familiar faces compared to unfamiliar faces may be explained by various factors. As we did not find any significant activity differences between the sessions, the poor ICCs cannot be explained by a significant loss of activity in session 2. However, substantial variation in brain activity over time, even within sessions, within these ROIs may account for low reliability estimates. The low stability of activity differences within each session is in accordance with this interpretation. The “task” in our study was a free‐viewing paradigm. Participants were asked to simply look at the stimuli. As a consequence, we did not control participants’ mental processes during the task. Thus, both within and between sessions, differences in mental state between participants and within participants over time are possible (e.g., due to variations in attention to and mental operations performed during the task). On the other hand, when participants have to perform a cognitive task during a face processing paradigm, task‐specific factors might affect how the brain processes the presented faces which may affect (condition differences) in FFA activity. For instance, in one earlier study, participants were asked to categorize faces for either their gender or their familiarity. In this study, the gender and the familiarity categorization task differentially affected the N170 component of the event‐related potential (Goffaux, Jemel, Jacques, Rossion, & Schyns, 2003), a component that has been related to face processing in the fusiform gyrus (Iidaka, Matsumoto, Haneda, Okada, & Sadato, 2006). In future research, including a cognitive task in the face processing paradigm used here may help to focus the attention of the participants on the presented faces. However, unintended effects on the processing of the presented faces by adding such a cognitive task should also be investigated.

Low reliabilities may also be caused by a low signal‐to‐noise ratio (SNR) of the fMRI data. However, we obtained stable ICCs in the face processing contrast (face vs. scrambled) for FFA activity, and significant effects obtained in session 1 were in accordance with expectations. Therefore, it is unlikely that the low reliabilities were due simply to excessive noise. That ICCs for FFA activity in the contrast faces versus scrambled stimuli were acceptable whereas ICCs obtained in the FFA, IFG, and the STG for the contrast familiar versus unfamiliar faces were not may instead be explained by the type of cognitive process reflected in these contrasts. With the contrast faces (unfamiliar and familiar) versus scrambled stimuli, basic face processing is investigated, and a large number of earlier studies report enhanced FFA activity in response to faces compared to non‐facial stimuli (e.g., Gauthier et al., 2000; Haxby et al., 2001). The contrast unfamiliar versus familiar faces target the brain processes involved in processing familiarity of the faces presented. Processing face familiarity seems to occur at a later processing stage (Eimer, 2000) and also appears to involve more diverse brain areas, with less consensus across studies concerning the areas involved (Natu & O'Toole, 2011). Nevertheless, changes in FFA, IFG, and STG activity in response to familiar faces compared to unfamiliar faces were reported in several earlier studies (Natu & O'Toole, 2011) in addition to our own (Heckendorf et al., 2016). Thus, although effects may not be as robust as changes in FFA activity related to basic face processing, FFA, IFG, and STG seem to play a role in processing face familiarity. Note also that low ICCs do not necessarily imply that group differences in brain activity in response to different types of stimuli (e.g., differences in response to familiar and unfamiliar faces) cannot be consistently significant. Rather, low ICCs imply that the size of the activity difference for individual participants is not stable over time. In all, more research is needed to further investigate the reliability of significant changes in brain activity related to familiarity processing.

Habituation of brain activity may also decrease ICCs. Because ICCs were consistently low, rather than acceptable when only the first few trials were included in the analyses and not when analyzing the entire task (expected when brain activity habituates within a session) or acceptable for the first but not the second session (expected when habituation occurs between sessions and/or during the second), we did not find strong evidence for habituation in the IFG, STG, and familiarity‐related information processing in the FFA (contrast: familiar vs. unfamiliar). In contrast, in V1, the lower reliability observed with an increasing number of trials as well as the lower within‐session reliability for session 2 than session 1 (particularly in right‐handers) may reflect habituation effects. We also obtained slight decreases in reliability of FFA activity for the face processing contrast (faces vs. scrambled) in session 2 compared to session 1, which could result from habituation effects. Habituation effects may for instance be caused by repeated exposure to only one unfamiliar‐looking and one familiar‐looking face per session. Including several familiar‐looking and unfamiliar‐looking faces may reduce habituation effects. Therefore, future studies may use a face processing paradigm with several different unfamiliar and familiar faces to investigate whether this increases fMRI test–retest reliabilities. Concerning the limited reliability of V1 activity elicited by the six combinations of primes and faces, it should also be noted that it was not possible to model hemodynamic responses to primes and faces separately, because primes and masks were presented very briefly and time‐locked to the presentation of the faces. Because, as a consequence, hemodynamic responses to these individual stimuli overlapped substantially, we modeled a single, summed hemodynamic response for the sequence mask‐prime‐mask‐face. In the contrasts with the fixation cross, activity in response to faces and primes, respectively, can therefore not be separated. Moreover, these contrasts do not isolate only the low‐level visual processes that take place in V1, which might negatively affect reliability. In contrast, the high‐level contrasts faces versus scrambled and familiar versus unfamiliar do isolate activity related specifically to processing faces and familiarity as the same primes were presented with each face. In future research, trials in which the prime and masks are omitted from the stimulus sequence could be included to enable separate modeling of neural responses to primes and faces, and estimation of the reliability of visual activity in response to primes and faces separately.

In their review, Bennett and Miller (2010) point out that fMRI reliability and the statistical power of fMRI experiments can both be enhanced by adding extra subjects and by increasing the length of the task. However, in our study, adding more trials did not substantially increase reliability. Habituation effects may further compromise the value of added trials. There may thus be a trade‐off between an enhanced signal resulting from extra trials, and increased noise with increasing task length due to habituation, subject fatigue or shifts in attention (Bennett & Miller, 2010). Thus, researchers need to carefully weigh these factors when deciding on the number of trials to include in an experiment, and further research investigating what constitutes and which factors affect the optimum number of trials is clearly necessary. Future research may also study habituation itself, by investigating when and how (e.g., at what rate, as a linear or non‐linear function of time) habitation takes place, and how to model habituation appropriately in fMRI analysis. This will enable estimation of effects of habituation on the reliability of fMRI data. With regard to differences in fMRI reliability between left‐handed and right‐handed participants, ICCs tended to be higher for left‐handed than right‐handed participants, but, apart from four exceptions (Supporting information), differences were not statistically significant. As our groups of left‐handed and right‐handed participants were relatively small, subtle differences in fMRI reliability between left‐handed and right‐handed individuals warrant attention in future research using larger samples. Note that because we did not find lower reliability for left‐handed participants, it seems clear that for tasks like the one used here reliability is no reason to justify the exclusion of left‐handed individuals from participation in a fMRI study.

Future research may also address some of the limitations of the current study. First, we examined fMRI reliability for one specific passive viewing paradigm. Although the introduction of a task may add to or alter information processing, a disadvantage of passive viewing may be that it is relatively difficult for participants to remain attentive and for researchers to monitor participants’ attentiveness. Studies examining the reliability of other research paradigms are badly needed. Moreover, future studies could examine fMRI reliability across a range of tasks (e.g., a memory, a motor and a visual task) to increase our understanding of how specific task characteristics may affect fMRI reliability when other relevant parameters, such as the scan procedure, are held constant. Second, participants in our study completed a face processing task embedded in a priming context. Although we did not find significant priming effects, we cannot exclude the possibility that the focus on priming affected test–retest reliability. Reliability studies using a face processing task without priming could confirm that the primes included in our study did not affect the ICCs obtained. Third, we only included child faces in our task. In future studies, stimuli may include individuals of varying ages (younger and older children as well as adults) to examine whether stimulus age affects test–retest reliability. In addition, behavioral indicators of face processing, such as participants’ memory for faces, could be included to examine if individual differences in capacities for face processing may influence reliability. Fourth, with 23 min, our task was relatively long. In future studies, the task could be split into several runs with short breaks in between, to examine whether this may increase reliabilities. In addition, the time span between sessions could be varied to systematically investigate how the time span between sessions affects test–retest reliability, as it has been suggested that longer time intervals between sessions are related to lower test–retest reliability (Bennett & Miller, 2010). Future research may also investigate whole‐brain ICCs to gain further insight into the reliability of both global and local indices of brain activity.

Finally, our sample only included female university students because of concerns for sample size and homogeneity, and the results may therefore not be generalizable to other populations (e.g., men, clinical groups). Large‐sample studies including, and comparing, both males and females are obviously welcome. Few have investigated fMRI reliability in clinical samples, but the existing studies indicate a lower fMRI reliability in clinical samples compared to healthy controls (see for a review Bennett & Miller, 2010). It would be interesting to use our face processing paradigm in individuals showing aberrant responses to social stimuli such as faces and examine test–retest reliability of fMRI activity among these individuals. To increase our understanding of the neurological deficits underlying deviant responses to faces, such as those reported in individuals with autism spectrum disorder, it is essential that we can reliably measure face processing in these individuals.

In conclusion, the current study showed relatively low fMRI reliability, with the exception of FFA activity related to face processing. This suggests that the paradigm used in this study, and perhaps fMRI more generally, is not ideally suited to study individual differences in brain activity. Low ICCs for fMRI data seem to be no exception. Although Bennett and Miller (2010) computed an average ICC of 0.50 across multiple fMRI reliability studies, ICCs varied substantially across ROIs and contrasts examined in individual studies (see also Herting et al., 2017, for similar findings in developmental samples). In addition, poor ICCs were found in several earlier studies focusing on face processing that examined fMRI reliability (e.g., Lipp et al., 2014; Van der Bulk et al., 2013). Also, some of the studies included in the meta‐analysis of Bennett and Miller (2010) examined very basic processes, such as motor processes, which has probably led to a higher average ICC. Based on the poor reliability values obtained in our and several other fMRI studies, it is important to look toward factors that may increase the reliability of fMRI measurements. Technical improvements of the MR hardware and software packages used to analyze the MRI data acquired remain desirable to enhance the progress of neuroimaging research. In addition, limited reliability stresses the need for larger samples in fMRI studies, as the associated measurement error in smaller samples elevates the risk of non‐reproducible group results. However, larger samples can never compensate for extremely low reliability. Moreover, the fact that the validity of a measurement is limited by its reliability (Shrout, 1998) makes the search for reliable fMRI assessments even more urgent.
