# Supplementary material for: Neural responses to children’s faces: Test–retest reliability of structural and functional MRI
Source: Brain Behav. 2019 Feb 9;9(3):e01192. doi: 10.1002/brb3.1192 (PMC6422824; doi:10.1002/brb3.1192)
Supplement: Supplementary file 1 [file BRB3-9-e01192-s001.docx]

**Supplementary Materials**

**Test-retest reliability, structural MRI**

**Table S1.** MRI volumetric measures of the structural analyses for the whole sample (N = 41) and test-retest reliability for the whole sample and for left-handed (N = 20) and right-handed (N = 21) participants separately.

| Area | Volume s1 in ml | | Volume s2 in ml | | ICCs | | | Fisher’s r to z | |
| --- | --- | --- | --- | --- | --- | --- | --- | --- | --- |
|  | Mean | SD | Mean | SD | Total | Left-handed | Right-handed | Z | p |
| Gray matter | 585.06 | 41.88 | 587.30 | 42.14 | 0.98 | 0.96 | 0.99 | -1.68 | 0.09 |
| White matter | 617.67 | 49.57 | 614.40 | 48.08 | 0.97 | 0.98 | 0.97 | 0.87 | 0.38 |
| Left amygdala | 1.40 | 0.15 | 1.41 | 0.16 | 0.88 | 0.94 | 0.81 | 1.84 | 0.07 |
| Right amygdala | 1.29 | 0.20 | 1.28 | 0.24 | 0.80 | 0.74 | 0.85 | -0.85 | 0.40 |
| Left thalamus | 8.06 | 0.58 | 8.07 | 0.54 | 0.96 | 0.97 | 0.95 | 0.73 | 0.47 |
| Right thalamus | 7.67 | 0.54 | 7.63 | 0.54 | 0.93 | 0.93 | 0.92 | 0.33 | 0.74 |

*Structural MRI data was available for one of the participants who provided no usable fMRI data.

**Test-retest reliability, a-priori defined ROIs**

Reliabilities obtained for maximum values within the a-priori defined ROIs were comparable to the test-retest reliabilities established for maximum values within the functional ROIs (see Table S2). Reliabilities for the contrast familiar vs. unfamiliar were poor for the whole sample, and for left-handed and right-handed participants separately, in all ROIs (FFA, IFG, STG; -.33 ≤ ICC ≤ .35, with the exception of FFA activity for left-handed participants ICC= .44 ). FFA activity related to face processing (contrast: face vs. scrambled) was fair to good both across the entire sample and for left- and right-handed participants separately (.41 ≤ ICC ≤ .62). For V1, reliability was poor for all contrasts vs. fixation cross for the entire sample (.24 ≤ ICC ≤.36) and for right-handed participants (.08 ≤ ICC ≤ .20). For left-handed participants reliability was fair (.40 ≤ ICC ≤ .56; with the exception of NeutralUnfamiliar vs. fixation cross: ICC = .29). Similar to the results obtained for maximum values within the functional masks, increasing the number of trials did not clearly increase reliability values, and even seemed associated with decreasing reliabilities for V1 (note the usually fair reliabilities for 78 trials, .39 ≤ ICC ≤ 51). In general, test-retest reliabilities for left-handed participants were slightly higher than for right-handed participants, but differences were not significant.

**Table S2.** Test-retest reliabilities for the a-priori defined masks with maximum values for the whole sample for the first third (78 trials N= 42^1^), the first two thirds of the task (156 trials N= 42^1^), and the complete task (234 trials N = 41), and for left- (N= 20) and right-handed (N= 21) participants separately.

| ROI | Contrast | Number of trials | | | Left-handed | Right-handed | Fisher’s r to z | |
| --- | --- | --- | --- | --- | --- | --- | --- | --- |
|  |  | 78 | 156 | 234 |  |  | Z | p |
| V1 | ThreatFamiliar vs. fix | 0.39 | 0.36 | 0.36 | 0.56 | 0.12 | 1.49 | 0.14 |
|  | ThreatUnfamiliar vs. fix | 0.40 | 0.39 | 0.34 | 0.48 | 0.14 | 1.12 | 0.26 |
|  | ThreatScrambled vs. fix | 0.51 | 0.37 | 0.34 | 0.51 | 0.14 | 1.24 | 0.22 |
|  | NeutralFamiliar vs. fix | 0.47 | 0.47 | 0.30 | 0.47 | 0.08 | 1.29 | 0.20 |
|  | NeutralUnfamiliar vs. fix | 0.44 | 0.40 | 0.24 | 0.29 | 0.20 | 0.27 | 0.79 |
|  | NeutralScrambled vs. fix | 0.44 | 0.28 | 0.27 | 0.40 | 0.11 | 0.96 | 0.34 |
| FFA | Familiar vs. Unfamiliar | 0.27 | 0.37 | 0.35 | 0.44 | 0.23 | 0.70 | 0.48 |
|  | Face vs. Scrambled | 0.50 | 0.58 | 0.54 | 0.41 | 0.62 | -0.82 | 0.41 |
| IFG | Familiar vs. Unfamiliar | 0.16 | 0.03 | -0.25 | -0.22 | -0.30 | 0.24 | 0.81 |
| STG | Familiar vs. Unfamiliar | 0.06 | -0.11 | -0.10 | -0.05 | -0.33 | 0.87 | 0.38 |

^1^For one participant, data was only available for the first and the second part of the task, since this participant fell asleep during the third part.

fix = fixation cross

**Test-retest reliability, mean and median cope values**

As shown in Tables S3 and S4, test-retest reliabilities for mean and median values within the functionally defined ROIs were lower than reliability scores obtained for maximum values. Good test-retest reliabilities were obtained only for activity related to face processing within the FFA (.54 ≤ ICC ≤ .72 [contrast face vs. scrambled]). All other ROIs and contrasts showed poor reliability (ICCs ≤ .39, with three exceptions among left-handed participants: ICC = .54 [median, IFG] ICC = .51 [mean, IFG], ICC = .43 [mean, FFA, familiar vs. unfamiliar]) Comparable to the results for maximum values, ICCs did not consistently increase with increasing numbers of trials and ICCs obtained for 78, 156, and 234 trials differed only slightly from each other (see Tables S3 and S4). Again, test-retest reliabilities for left-handed participants were higher than for right-handed participants, but after correcting for multiple testing, differences were not significant.

**Table S3.** Test-retest reliabilities for the functional masks with mean values for the whole sample for the first third (78 trials N= 42^1^), the first two thirds of the task (156 trials N= 42^1^), and the complete task (234 trials N = 41), and for left- (N= 20) and right-handed (N= 21) participants separately.

| ROI | Contrast | Number of trials | | | Left-handed | Right-handed | Fisher’s r to z | |
| --- | --- | --- | --- | --- | --- | --- | --- | --- |
|  |  | 78 | 156 | 234 |  |  | Z | p |
| V1 | ThreatFamiliar vs. fix | 0.11 | 0.09 | 0.05 | 0.11 | -0.01 | 0.36 | 0.72 |
|  | ThreatUnfamiliar vs. fix | 0.12 | 0.05 | 0.09 | 0.17 | 0.00 | 0.53 | 0.60 |
|  | ThreatScrambled vs. fix | 0.28 | 0.10 | 0.09 | 0.16 | 0.02 | 0.42 | 0.68 |
|  | NeutralFamiliar vs. fix | 0.11 | 0.09 | 0.07 | 0.10 | 0.04 | 0.16 | 0.87 |
|  | NeutralUnfamiliar vs. fix | 0.14 | 0.14 | 0.11 | 0.20 | 0.04 | 0.48 | 0.63 |
|  | NeutralScrambled vs. fix | 0.13 | 0.10 | 0.07 | 0.12 | 0.02 | 0.29 | 0.77 |
| FFA | Familiar vs. Unfamiliar | 0.19 | 0.23 | 0.21 | 0.43 | -0.09 | 1.60 | 0.11 |
|  | Face vs. Scrambled | 0.54 | 0.61 | 0.65 | 0.56 | 0.71 | -0.76 | 0.45 |
| IFG | Familiar vs. Unfamiliar | 0.07 | 0.07 | 0.02 | 0.51 | -0.23 | 2.34 | 0.02* |
| STG | Familiar vs. Unfamiliar | -0.02 | 0.15 | 0.23 | 0.27 | 0.16 | 0.35 | 0.73 |

^1^For one participant, data was only available for the first and the second part of the task, since

this participant fell asleep during the third part.

*Difference was not significant after applying the Benjamini-Hochberg procedure to correct for multiple testing.

fix = fixation cross

**Table S4.** Test-retest reliabilities for the functional masks with median values of the whole sample for the first third (78 trials N= 42^1^), the first two thirds of the task (156 trials N= 42^1^), and the complete task (234 trials N = 41), and for left- (N= 20) and right-handed (N= 21) participants separately.

| ROI | Contrast | Number of trials | | | Left-handed | Right-handed | Fisher’s r to z | | |
| --- | --- | --- | --- | --- | --- | --- | --- | --- | --- |
|  |  | 78 | 156 | 234 |  |  | | Z | p |
| V1 | ThreatFamiliar vs. fix | 0.11 | 0.10 | 0.04 | 0.10 | 0.00 | | 0.32 | 0.75 |
|  | ThreatUnfamiliar vs. fix | 0.10 | 0.04 | 0.08 | 0.17 | 0.00 | | 0.50 | 0.62 |
|  | ThreatScrambled vs. fix | 0.22 | 0.07 | 0.05 | 0.10 | 0.00 | | 0.30 | 0.76 |
|  | NeutralFamiliar vs. fix | 0.07 | 0.09 | 0.07 | 0.08 | 0.06 | | 0.05 | 0.96 |
|  | NeutralUnfamiliar vs. fix | 0.10 | 0.14 | 0.13 | 0.23 | 0.05 | | 0.53 | 0.60 |
|  | NeutralScrambled vs. fix | 0.05 | 0.09 | 0.05 | 0.07 | 0.03 | | 0.10 | 0.92 |
| FFA | Familiar vs. Unfamiliar | 0.23 | 0.20 | 0.17 | 0.39 | -0.11 | | 1.53 | 0.13 |
|  | Face vs. Scrambled | 0.60 | 0.63 | 0.66 | 0.57 | 0.72 | | -0.80 | 0.42 |
| IFG | Familiar vs. Unfamiliar | 0.10 | 0.06 | 0.04 | 0.54 | -0.22 | | 2.42 | 0.02* |
| STG | Familiar vs. Unfamiliar | -0.01 | 0.10 | 0.25 | 0.28 | 0.17 | | 0.34 | 0.73 |

^1^For one participant, data was only available for the first and the second part of the task, since

this participant fell asleep during the third part.

*Difference was not significant after applying the Benjamini-Hochberg procedure to correct for multiple testing.

fix = fixation cross

**Test-retest reliability for consistency**

Table S5 displays test-retest reliabilities for consistency, calculated for maximum cope values within functional ROIs. ICCs for consistency were generally comparable to ICCs for absolute agreement (see Table S5). For the contrast familiar vs. unfamiliar (FFA, IFG, STG), ICCs for consistency were poor (-.16 ≤ ICC ≤ .34), with the exception of fair reliability for FFA activity obtained for left-handed participants (ICC = .53). FFA activity related to face processing (contrast: face vs. scrambled) showed good test-retest reliability (.65 ≤ ICC ≤ .74). For V1, ICCs for right-handed participants were poor (.02 ≤ ICC ≥ .18), but ICCs for left-handed participants were fair to excellent (.52 ≤ ICC ≤ .84) and thus somewhat higher than ICCs obtained for absolute agreement. Across the entire sample, ICCs were poor to fair for V1 activity (.28 ≤ ICC ≥ .55). ICCs were generally higher for left-handed than for right-handed participants, with significant differences obtained in V1 for the contrasts ThreatFamilar vs. fixation cross, ThreatScrambled vs. fixation cross, and NeutralUnfamiliar vs. fixation cross after correcting for multiple testing.

**Table S5.** Test-retest reliabilities for maximum values of the whole sample for the first third (78 trials N= 42^1^), the first two thirds of the task (156 trials N= 42^1^), and the complete task (234 trials N = 41) , and for left- (N= 20) and right-handed (N= 21) participants separately (234 trials).

| ROI | Contrast | Number of trials | | | Left-handed | Right-handed | Fisher’s r to z | |
| --- | --- | --- | --- | --- | --- | --- | --- | --- |
|  |  | 78 | 156 | 234 |  |  | Z | p |
| V1 | ThreatFamiliar vs. fix | 0.44 | 0.41 | 0.43 | 0.77 | 0.07 | 2.81 | 0.01** |
|  | ThreatUnfamiliar vs. fix | 0.41 | 0.43 | 0.38 | 0.64 | 0.06 | 2.06 | 0.04* |
|  | ThreatScrambled vs. fix | 0.55 | 0.49 | 0.53 | 0.84 | 0.18 | 3.07 | 0.00** |
|  | NeutralFamiliar vs. fix | 0.48 | 0.50 | 0.34 | 0.65 | 0.02 | 2.23 | 0.03* |
|  | NeutralUnfamiliar vs. fix | 0.45 | 0.46 | 0.28 | 0.52 | 0.18 | 1.17 | 0.24 |
|  | NeutralScrambled vs. fix | 0.49 | 0.38 | 0.44 | 0.82 | 0.10 | 3.12 | 0.00** |
| FFA | Familiar vs. Unfamiliar | 0.13 | 0.25 | 0.29 | 0.53 | 0.04 | 1.63 | 0.10 |
|  | Face vs. Scrambled | 0.65 | 0.65 | 0.71 | 0.66 | 0.74 | -0.47 | 0.64 |
| IFG | Familiar vs. Unfamiliar | 0.06 | 0.11 | 0.01 | 0.27 | -0.16 | 1.3 | 0.19 |
| STG | Familiar vs. Unfamiliar | 0.12 | 0.08 | 0.18 | 0.34 | -0.04 | 1.17 | 0.24 |

^1^For one participant, data was only available for the first and the second part of the task, since this participant fell asleep during the third part.

fix = fixation cross

*Difference was not significant after applying the Benjamini-Hochberg procedure to correct for multiple testing.

**Difference was significant after applying the Benjamini-Hochberg procedure to correct for multiple testing.

**Within-session reliability**

Table S6 presents within-session reliabilities for maximum values (functional ROIs). In session 1, reliability of V1 activity was fair to excellent across the entire sample (.49 ≤ ICC ≤ .77) and for left- and right-handed participants separately (.47 ≤ ICC ≤ .80). In session 2, reliability of V1 activity was fair to excellent for left-handed participants (.41 ≤ ICC ≤ .77), but poor for right-handed participants (ICC ≤ .31, except NeutralScrambled vs. fixation: ICC = .46), resulting in poor to good reliabilities across the entire sample (.34 ≤ ICC ≤ .61). Reliability of FFA activity related to face processing (contrast face vs. scrambled) was clearly higher in session 1 (.51 ≤ ICC ≤ .67; fair to good) compared to session 2 (.24 ≤ ICC ≤ .49; poor to fair). For the contrast familiar vs. unfamiliar, we obtained poor reliability values in both sessions for all ROIs (session 1: .01 ≤ ICC ≤ .34; except for IFG activity in right-handed participants, ICC= .42; session 2: -.14 ≤ ICC ≤ .25). With respect to handedness, we did not obtain systematic differences in ICC values between left-handed and right-handed participants in session 1. However, in session 2 reliabilities were systematically higher for left-handed than for right-handed participants, although differences were significant only for V1 activity for the contrast NeutralUnfamiliar vs. fixation cross (*p* < .01) after correcting for multiple testing using the Benjamini-Hochberg procedure.

**Table S6.** Test-retest reliabilities for the functional masks with max values for the whole group, and for left- (N = 20) and right-handed (N= 21) participants separately within session 1 and within session 2.

| ROI | Contrast | Session 1 | | | Session 2 | | |
| --- | --- | --- | --- | --- | --- | --- | --- |
|  |  | Whole sample | Left-handed | Right-handed | Whole sample | Left-handed | Right-handed |
| V1 | ThreatFamiliar vs. fix | 0.65 | 0.64 | 0.66 | 0.34 | 0.44 | 0.24 |
|  | ThreatUnfamiliar vs. fix | 0.49 | 0.52 | 0.48 | 0.45 | 0.73* | 0.15 |
|  | ThreatScrambled vs. fix | 0.69 | 0.64 | 0.74 | 0.52 | 0.73 | 0.31 |
|  | NeutralFamiliar vs. fix | 0.67 | 0.77 | 0.58 | 0.35 | 0.41 | 0.28 |
|  | NeutralUnfamiliar vs. fix | 0.61 | 0.47 | 0.77 | 0.38 | 0.77** | 0.13 |
|  | NeutralScrambled vs. fix | 0.77 | 0.72 | 0.80 | 0.61 | 0.77 | 0.46 |
| FFA | Familiar vs. Unfamiliar | 0.16 | 0.11 | 0.22 | 0.08 | 0.13 | 0.02 |
|  | Face vs. Scrambled | 0.60 | 0.51 | 0.67 | 0.36 | 0.24 | 0.49 |
| IFG | Familiar vs. Unfamiliar | 0.34 | 0.17 | 0.42 | 0.00 | 0.13 | -0.12 |
| STG | Familiar vs. Unfamiliar | 0.17 | 0.01 | 0.28 | 0.13 | 0.25 | -0.14 |

Effects for Handedness are calculated with Fisher’s r to z transformation.

*Difference was not significant after applying the Benjamini-Hochberg procedure to correct for multiple testing.

**Difference was significant after applying the Benjamini-Hochberg procedure to correct for multiple testing.
